# A metabolome-wide characterization of the diabetic phenotype in ZDF rats and its reversal by pioglitazone

**DOI:** 10.1371/journal.pone.0207210

**Published:** 2018-11-27

**Authors:** Thomas J. Jönsson, Hans-Ludwig Schäfer, Andreas W. Herling, Mark Brönstrup

**Affiliations:** 1 Metabolon Inc, Durham, North Carolina, United States of America; 2 Sanofi R&D, Industriepark Hoechst, Frankfurt, Germany; 3 Helmholtz Centre for Infection Research and German Center for Infection Research (DZIF), Braunschweig, Germany; University of Glasgow, UNITED KINGDOM

## Abstract

Type 2 diabetes (T2D) is a complex metabolic disease associated with alterations in glucose, lipid and protein metabolism. In order to characterize the biochemical phenotype of the Zucker diabetic fatty (ZDF) rat, the most common animal model for the study of T2D, and the impact of the insulin sensitizer pioglitazone, a global, mass spectrometry-based analysis of the metabolome was conducted. Overall, 420 metabolites in serum, 443 in the liver and 603 in the intestine were identified at study end. In comparison to two control groups, obese diabetic ZDF rats showed characteristic metabolic signatures that included hyperglycemia, elevated β-oxidation, dyslipidemia—featured by an increase in saturated and monounsaturated fatty acids and a decrease of medium chain and of polyunsaturated fatty acids in serum–and decreased amino acid levels, consistent with their utilization in hepatic gluconeogenesis. A 13-week treatment with the PPARγ agonist pioglitazone reversed most of these signatures: Pioglitazone improved glycemic control and the fatty acid profile, elevated amino acid levels in the liver, but decreased branched chain amino acids in serum. The hitherto most comprehensive metabolic profiling study identified a biochemical blueprint for the ZDF diabetic model and captured the impact of genetic, nutritional and pharmacological perturbations. The in-depth characterization on the molecular level deepens the understanding and further validates the ZDF rat as a suitable preclinical model of diabetes in humans.

## Introduction

The number of people suffering from type 2 diabetes (T2D) and its complications has risen significantly in the past decades in all continents of the globe, rendering the disease a major threat for public health.[1,2] Type 2 diabetes is a slowly progressing, multifactorial disease defined by hyperglycemia that exhibits multiple alterations in glucose, lipid and protein metabolism. A hallmark of the early stages of T2D is insulin resistance, i.e. an impaired insulin-mediated cellular uptake of glucose, while advanced stages are characterized by a dysfunction of pancreatic β-cells, leading to insufficient insulin secretion and overt hyperglycemia.[3–6] Defined animal models that mirror the pathogenesis in humans have played a valuable role in studying the complex manifestation of T2D and for assessing new treatment modalities. The male Zucker diabetic fatty (ZDF) rat with fa/fa-genotype is commonly used, as it spontaneously becomes obese and hyperglycemic within the first few months of life due to a leptin receptor defect and a genetically reduced insulin promoter activity.[7–10] Female ZDF rats with fa/fa-genotype similarly become obese and insulin-resistant, but do not progress to hyperglycemia, except when fed a high-fat diet. Diabetic ZDF rats were found to have an insufficient insulin secretion ability that cannot compensate for the insulin resistance associated with their obesity.[11,12] Although the mechanism(s) responsible for the failure of β-cell mass expansion have not been fully elucidated, such a defect may be secondary to the gluco- and lipotoxicity associated with the diabetic state.[7,9,13]

Multiple studies from our and other groups have characterized the disease phenotype in ZDF rats and the impact of pharmacological treatments by established clinical biomarkers of diabetes, dyslipidemia and their micro- and macrovascular complications.[14,15] The objective of this study was to obtain a more comprehensive picture of the metabolic changes that are associated with the diabetic state in the ZDF rat. For this purpose, the metabolome of female ZDF (fa/fa) rats that were diabetic following a high fat diet was compared to that of isogenic counterparts that obtained a normal chow and were therefore nondiabetic. The impact of the homozygous fa/fa-genotype was captured by comparing fa/fa rats to Fa/? controls, with both groups receiving an identical, normal chow. Finally, the effects of a pharmacological intervention with the marketed insulin sensitizer pioglitazone on the metabolome were investigated. Only female animals were selected for the study for the following reasons: (i) The disease status of male animals is very heterogeneous within the first 3 month of life during that age, and the variability between animals and groups is high. (ii) As the treatment of male animals with pioglitazone before the onset of diabetes (at an age of ca. 6 weeks) delays the onset and the progression of diabetes, treated non-diabetic would be compared with untreated diabetic male animals. Thus, we have decided to investigate only female animals in order to probe a compound effect in a stable animal model.

Pioglitazone and rosiglitazone are the two most prominent, yet differentiated members of the class of thiazolidinediones (TZDs) which exert insulin-sensitizing effects, a re-distribution of body fat, an increase in lipid uptake and mitochondrial β-oxidation in adipose tissue, and other metabolic effects through activation of the nuclear receptor peroxisome proliferator-activated receptor gamma (PPARγ), the major transcriptional regulator in adipocytes.[16–18] While most metabolomics studies on TZD’s focused on rosiglitazone,[19–22] pioglitazone specifically was investigated by Kus *et al*., who have quantified 163 metabolites in a targeted manner to compare it *vs*. rosiglitazone in combination with n-3 long-chain polyunsaturated fatty acids in C57BL/6N mice.[23,24] Radonic *et al*. used a GC/MS analysis to detect changes of more than 100 identified metabolites in LDLR -/- mice on a high fat diet following treatment with 10 drugs, including pioglitazone.[25] The effect of pioglitazone on fatty acids of adipocytes isolated from insulin-resistant rats has been recently reported by Sato *et al*.[26] Purine metabolites in cardiac tissues from pioglitazone-treated rats have been investigated by NMR spectroscopy.[27] Metabolic studies in humans treated with pioglitazone focused on effects on amino acid profiles,[28] metabolic indicators of maturation of high-density lipoproteins (HDL),[29] or metabolite changes in women with polycystic ovary syndrome (PCOS).[30]

The present study aimed at characterizing changes of the metabolome in the ZDF rat model that are associated with genetic (fa/fa vs. Fa/?) nutritional (normal chow *vs*. high fat diet) and pharmacological (untreated *vs*. pioglitazone) perturbations. For this purpose, a combination of gas chromatography (GC)- and liquid chromatography (LC)-coupled mass spectrometry was applied to analyze the serum, liver, and intestine metabolome. With 420 identified biochemicals in serum, 443 in liver and 603 in intestine, the study represents the most comprehensive analysis of metabolome effects in the ZDF model.

## Materials and methods

### Animal studies and interventions

A study-specific approval for the Research was obtained. The study was conducted under the following legal frame: Tierversuchsanzeige-Nr.: HMR 4b Anz. 38, Methode 4, which has been approved by the Regierungspräsidium Darmstadt (Hessen, Germany). The studies were performed under the terms of the German Animal Protection Law and in accordance with the Sanofi Company Charter on the Humane Care and Use of Laboratory Animals. The institution is AAALAC accredited.

Female ZDF rats (ZDF-Lepr^fa^/Crl) were obtained from Charles River Kissleg, Germany at an age of 8 weeks. Obese (genotype: fa/fa) rats or lean (genotype (Fa/?) rats were used after one week acclimation with an age of 9 weeks at start of the study. Animals were housed under controlled temperature (21–23°C) and humidity (55%) in Macrolon type 4 cages (2 animals per cage) with a 12 h light-dark-cycle (light phase, 6:00–18:00) with *ad libitum* access to drinking water. Animals were divided into 4 groups (6 animals/group) with group 1 (Fa/?) and 2 (fa/fa) receiving a normal chow (Teklad standard rodent diet T.2014.12). Groups 3 (fa/fa) and 4 (fa/fa) received a high fat diet and were treated with vehicle control, or pioglitazone (Actos®) daily (10 mg/kg p.o.), for 13 weeks, respectively. The rats in groups 3 and 4 were on normal chow for 9 weeks after birth, thereafter for 10 weeks on TD97366, which was followed by 3 weeks on D12468, because the latter enhanced the progression to diabetes. TD97366 contained 68% calories from fat, and D12468 contained 48% calories from fat.

### Serum, liver and intestine sample preparation

Blood samples for glucose and insulin analysis were taken from tail tip without anesthesia. Blood for other serum parameters was drawn from the retroorbital vein plexus under isoflurane (CP Pharma, Burgdorf, Germany) oxygen/nitric oxide anesthesia (3.5%, 2:1). Animals were anesthetized in a gas box. All serum samples were taken at the beginning of the study and at study end (day 92, week 13), with the exception of insulin data that are reported for samples taken at day 71. At the end of the study the animals were sacrificed by bleeding from the abdominal aorta after laparotomy under deep Isoflurane oxygen/nitric oxide anesthesia. In all cases, treatment was applied between 7:30 and 8:30 a.m., and blood samples were drawn 1 hour after treatment. Blood was collected in Mikrovette 500 Z-Gel cups, at the study end in S-Monovette 9ml Z-Gel vials containing a clotting activator (Sarstedt, Germany), stored 30 minutes at room temperature, and centrifuged thereafter at 2000 g for 10 minutes in a refrigerated centrifuge at 4–8°C. The samples have been stored in a refrigerator at -80°C.

Immediately after bleeding from abdominal aorta, the liver and jejunum were dissected. Samples of 50–10 mg liver tissue, representing a part of the liver, were shock frozen in liquid nitrogen and stored at -80°C. A 20 cm segment of the jejunum was longitudinal opened, softly stripped, rinsed in cold PBS buffer, shock frozen in liquid nitrogen and stored at -80°C. The jejunum section of the intestine was selected to investigate possible incretin related effects.

### Clinical chemistry

Most standard biomarkers of clinical chemistry were determined on a Roche Cobas 6000 analyzer using the respective Roche clinical chemistry kits for human diagnostics. Free fatty acids were determined using a Wako kit adapted to the Cobas 6000. Liver cholesterol and liver triglycerides were determined on a Beckman AU680 analyzer using standard Beckman reagents. Blood glucose was determined using a Hengler Analytik reagent (hexokinase method) on a Beckman AU680 instrument. Insulin was determined using Mercodia Rat Insulin ELISA assay kits (High Range: 10-1145-12 / Ultrasensitive: 10–1250). Total GLP-1 was determined using the Mesoscale Total GLP-1 Assay Kit (K150JVC-2) and a sector S600 reader. Rat Adiponectin was determined using an ELISA kit from B-Bridge International (K-1002-1) For both assays, the Tecan Infinite F500 microplate reader was used. All assays were performed according to the instructions from the suppliers.

### Metabolomic profiling technology

#### Sample storage and preparation

Frozen serum samples were shipped under dry ice to Metabolon (Durham, NC) for metabolite analysis. A total of 72 samples, generated from 6 animals/group and 3 matrices (serum, liver, intestine) per animal at the end of the study, were analyzed. Metabolomic profiling was performed as described previously [31,32]. The tissues were mixed with water and homogenized with beads. Metabolites were extracted from 100 μL serum or 20 μg/ml homogenized tissue by the addition of cold methanol. The precipitated extract was split into four aliquots and dried under nitrogen. The samples were re-suspended in platform specific solutions before they were applied into the instruments. The untargeted metabolomic profiling platform employed for this analysis was based on a combination of three independent platforms: ultrahigh performance liquid chromatography/tandem mass spectrometry (UHPLC/MS/MS) optimized for basic species, UHPLC/MS/MS optimized for acidic species and gas chromatography/mass spectrometry (GC/MS) [31,32]. LC-MS was performed on a Waters ACQUITY ultra-performance liquid chromatography (UPLC) and a Thermo-Finnigan LTQ mass spectrometer, which consisted of an electrospray ionization (ESI) source and linear ion-trap (LIT) mass analyzer [31]. Samples destined for GC/MS analysis were derivatized under dried nitrogen using bistrimethyl-silyl-trifluoroacetamide (BSTFA). Samples were analyzed on a Thermo-Finnigan Trace DSQ fast-scanning single-quadrupole mass spectrometer using electron impact ionization at unit mass resolution [32]. Metabolites were identified by matching the ions’ chromatographic retention index and mass spectral fragmentation signatures with reference library entries created from authentic standard metabolites. For ions that were not covered by the standards, additional library entries were added based on their unique retention time and ion signatures. Peak ion counts for each compound in each sample were used for statistical analysis, resulting in the comparisons of relative concentrations. The data was not normalized. Peaks for each metabolite were quantified using area-under-the-curve for the ion counts. The statistical analyses were performed on the peak area data (ion counts). A given compound was reported from only one of the three platforms.

For quality control purposes, a number of internal standards were added to each experimental and process standard sample just prior to injection into the mass spectrometers. Because these standards were added to the samples immediately prior to injection into the instrument, this value reflects instrument variation. In addition, an aliquot of each experimental serum sample was pooled for the creation of “Client Matrix” (CMTRX) samples. These CMTRX samples were injected throughout the platform run and served as technical replicates, allowing us to determine the quantitative variability of all consistently detected biochemicals and overall process variability, and to monitor platform performance. A measure of the platform variability was determined by calculating the median relative standard deviation (RSD) for the internal standards and for the CMTRX samples. Median RSD’s of 8%, 8% and 6% were observed for the internal standards in intestine, serum and liver samples, respectively. For CMTRX samples, median RSD’s of 15%, 14% and 13% were observed in intestine, serum and liver samples, respectively. The results for the CMTRX and internal standards indicated that the platform produced data that met process specifications.

#### Statistical analysis

Welch’s two sample *t*-tests were used to analyze the data. For all analyses, missing values, if any, were imputed with the observed minimum for that particular compound. The statistical analyses were performed on natural log-transformed data. False-discovery rates (FDR) were computed because of the multiple comparisons with the q-value method to account for multiple comparisons according to Storey *et al*.[33]. The q-values were estimated with the R-package and listed for each metabolite in the S1 File [34].

The relative abundance of metabolites has been visualized in the data tables below with the following color code:

Green: indicates significant difference (p≤0.05) between the groups shown; GREEN indicates a ratio < 1; Red: indicates significant difference (p≤0.05) between the groups shown; RED indicates a ratio > 1; Light green: narrowly missed cutoff for significance; 0.05<p<0.10; light green indicates a ratio < 1; Light red: narrowly missed cutoff for significance; 0.05<p<0.10; light red indicates a ratio > 1; Non-colored text and cell: mean values are not significantly different for that comparison.

## Results and discussion

### The ZDF rat model

In order to characterize metabolic adaptations in the diabetic ZDF rat model, three groups of animals, aged 9 weeks at the start of the study, were compared to each other: Female ZDF rats with a homozygous fa/fa-genotype were compared to lean Fa/? controls; both groups received an identical, normal chow (NC) for 13 weeks. The obese (NC) group with fa/fa-genotype was compared to isogenic counterparts that became diabetic following a high fat (HF) diet. Clinical chemistry data and physiological characteristics of the groups at study start and study end are listed in Table 1. Blood glucose levels were almost unchanged in the Fa/? (study end: 5.84 mM vs. study start: 6.01 mM) and obese fa/fa group receiving normal chow (5.91 mM vs. 6.53 mM), but fa/fa animals under high fat diet developed hyperglycemia, as indicated by blood glucose levels of 19.7 mM. The treatment of fa/fa animals under high fat diet with pioglitazone led to a pronounced decrease of blood glucose levels to 6.37 mM. The high fat diet also led to a pronounced increase of body weight, serum and liver triglycerides, and the doubling of free fatty acid levels in serum. Insulin levels were equal between both fa/fa groups, but significantly higher compared to the Fa/? groups, indicating an insulin resistant state in the fa/fa genotype. The inclusion of the lean (Fa/?) control rats allowed to fully capture the effects of pioglitazone treatment, as demonstrated by changes of serum insulin, serum GLP-1, or triglycerides. The levels of these markers were reduced even to the level of lean (Fa/?) control rats, beyond that of obese (fa/fa) control rats on normal chow (NC). It was also associated with a gain in body weight and a pronounced increase of adiponectin that is characteristic for the PPARγ agonists.[35]

**Table 1:** Clinical chemistry and physiological data of treatment groups at study start and study end (week 13).

	start of study							end of study						
mean value S.E.M.	lean (NC)	obese (NC)	obese (HF)	obese (HF) + Pio	p- value lean (NC) vs obese (NC)	p- value obese (NC) vs obese (HF)	p- value obese (HF) vs obese (HF) + Pio	lean (NC)	obese (NC)	obese (HF)	obese (HF) + Pio	p- value lean (NC) vs obese (NC)	p- value obese (NC) vs obese (HF)	p- value obese (HF) vs obese (HF) + Pio
**food comsumption in g/d/animal**	**15.9**	**26.7**	**21.4**	**29.7**	**8.10E-01**	**2.38E-03**	**9.31E-01**	**12.7**	**19.8**	**16.3**	**10.3**	**3.6E-02**	**4.9E-04**	**9.9E-01**
	**0.24**	**0.29**	**0.49**	**0.58**	** **	** **	** **	**0.34**	**0.61**	**0.71**	**1.39**	** **	** **	** **
**body weight in g**	**178**	**280**	**284**	**290**	**4.00E-09**	**6.04E-01**	**5.98E-01**	**226**	**416**	**473**	**772**	**2.5E-12**	**4.6E-03**	**3.8E-07**
	**1.81**	**4.50**	**6.88**	**6.57**	** **	** **	** **	**2.69**	**4.94**	**14.27**	**20.01**	** **	** **	** **
**glucose in mM (B)**	**6.01**	**6.53**	**6.52**	**6.45**	**5.09E-02**	**9.72E-01**	**7.72E-01**	**5.84**	**5.91**	**19.70**	**6.37**	**8.0E-01**	**1.7E-04**	**2.0E-04**
	**0.15**	**0.19**	**0.09**	**0.22**	** **	** **	** **	**0.14**	**0.20**	**1.92**	**0.26**	** **	** **	** **
insulin in ng/mL (S)*	**0.43**	**6.32**	**7.09**	**10.7**	**3.50E-04**	**5.57E-01**	**6.63E-02**	**1.07**	**17.1**	**14.0**	**5.09**	**1.1E-03**	**4.4E-01**	**8.4E-03**
	**0.05**	**0.92**	**0.88**	**1.55**	** **	** **	** **	**0.21**	**3.02**	**2.48**	**0.37**	** **	** **	** **
**GLP-1 in pg/mL (S)**	**4.83**	**16.29**	**16.26**	**18.16**	**7.24E-04**	**9.88E-01**	**2.65E-01**	**3.88**	**16.09**	**13.89**	**4.31**	**5.1E-04**	**3.8E-01**	**3.1E-04**
	**0.78**	**2.13**	**0.92**	**1.35**	** **	** **	** **	**0.56**	**2.10**	**1.16**	**1.41**	** **	** **	** **
**cholesterol in mM (B)**	**1.97**	**2.76**	**2.79**	**2.81**	**2.42E-05**	**7.88E-01**	**8.15E-01**	**2.59**	**3.33**	**3.57**	**4.49**	**1.3E-04**	**3.7E-01**	**1.0E-02**
	**0.09**	**0.09**	**0.06**	**0.08**	** **	** **	** **	**0.10**	**0.10**	**0.23**	**0.19**	** **	** **	** **
**triglycerides in mM (B)**	**1.10**	**16.1**	**14.1**	**13.8**	**3.65E-06**	**1.90E-01**	**8.80E-01**	**1.51**	**17.3**	**17.3**	**2.82**	**3.3E-07**	**9.9E-01**	**5.0E-05**
	**0.20**	**1.21**	**0.85**	**1.77**	** **	** **	** **	**0.16**	**0.92**	**1.66**	**0.10**	** **	** **	** **
**free fatty acids in mM (B)**	**0.26**	**0.36**	**0.33**	**0.34**	**8.64E-03**	**3.43E-01**	**6.49E-01**	**0.28**	**0.56**	**1.05**	**0.74**	**1.1E-05**	**2.2E-04**	**6.0E-02**
	**0.02**	**0.02**	**0.01**	**0.02**	** **	** **	** **	**0.01**	**0.03**	**0.08**	**0.12**	** **	** **	** **
**adiponectin in ng/mL (S)**	**9348**	**13425**	**12770**	**12576**	**2.07E-03**	**4.89E-01**	**8.26E-01**	**4791**	**5523**	**6937**	**20000**	**1.9E-01**	**5.2E-02**	**1.4E-08**
	**755**	**774**	**492**	**706**	** **	** **	** **	**148**	**493**	**446**	**0**	** **	** **	** **
**liver weight in g**	** **	** **	** **	** **	** **	** **	** **	**7.03**	**16.2**	**20.6**	**15.3**	**4.5E-12**	**2.8E-05**	**8.6E-05**
	** **	** **	** **	** **	** **	** **	** **	**0.13**	**0.25**	**0.55**	**0.67**	** **	** **	** **
**liver cholesterol in mg/g**	** **	** **	** **	** **	** **	** **	** **	**2.19**	**2.02**	**2.47**	**3.16**	**6.9E-04**	**6.4E-04**	**2.1E-01**
	** **	** **	** **	** **	** **	** **	** **	**0.02**	**0.03**	**0.08**	**0.48**	** **	** **	** **
**liver triglycerides in mg/g**	** **	** **	** **	** **	** **	** **	** **	**5.39**	**43.1**	**106**	**95.2**	**2.4E-03**	**2.4E-04**	**4.8E-01**
	** **	** **	** **	** **	** **	** **	** **	**0.33**	**8.15**	**9.78**	**11.34**	** **	** **	** **

Data are given as mean values (S.E.M in second line). (B): from blood; (S): from serum.

*Insulin data were obtained at day 71. p-values were calculated by student’s t-test.

### Metabolome analysis

At the end of the study, the metabolome of serum and of two organs, the liver and the intestine, was recorded by mass spectrometry in the three untreated groups of ZDF rats.

Overall, 420 biochemicals in serum, 443 in liver and 603 in intestine could be detected (S1 File, Tables A-F). This total corresponds to 298, 289 and 377 biochemicals with known structures in the three respective matrices. The remaining identified components (122 in serum, 154 in the liver, and 226 in the intestine) represented distinct chemical entities (i.e. a single molecule of discrete molecular formula) with unknown chemical structures.

A comparison between the three untreated groups of ZDF rats was made on the basis of relative abundances of metabolites. Relative ratios between the homozygous fa/fa-genotype and the lean Fa/? control are expressed as obese (NC)/lean (NC). In addition, the obese (NC) group with fa/fa-genotype was compared to their isogenic counterparts that received a high fat (HF) diet based on the intensity ratios obese (HF)/obese (NC) for each metabolite.

A summary of the numbers of biochemicals that were observed to be significantly different (*p*≤0.05, no *q*-value cut-off) by *t*-test analysis is given in Table 2. Also listed are the directional changes of these biochemicals and the estimated false discovery rate (FDR or *q*-value) for the subset of compounds with *p*≤0.05.

**Table 2:** Overview on number of altered biochemicals and directional changes between the treatment groups.

Summary of altered biochemicals		Ob (NC) /	Ob (HF) /	Pio (HF) /
		Lean (NC)	Ob (NC)	Ob (HF)
	Biochemicals with p≤0.05	97	175	170
Intestine	Biochemicals (↑/↓)	38/59	124/51	114/56
	q-value	0.24	0.08	0.05
	Biochemicals with p≤0.05	166	216	222
Serum	Biochemicals (↑/↓)	121/45	71/145	86/136
	q-value	0.07	0.04	0.03
	Biochemicals with p≤0.05	224	195	176
Liver	Biochemicals (↑/↓)	100/124	55/140	73/103
	q-value	0.04	0.05	0.05

The number of upregulated biochemicals is shown first (before the slash sign), and the number of downregulated biochemicals is given after the slash sign. The q-value is the average value across all regulated metabolites in the given group.

As indicated by the sizeable numbers of biochemicals that were observed to be significantly different between the various treatment groups, the genetic, nutritional and pharmacological perturbations had a significant impact on the metabolome. For example, 51% of serum metabolites changed upon feeding a high-fat diet to isogenic animals, and 29% of liver metabolites were up- or downregulated upon treatment with pioglitazone.

A data set with all unprocessed area counts for all features in all samples is provided in the supplement. A limitation of the study is that the raw instrument data are not made available, as they contain trade secrets of Metabolon. However, in addition to new and unprecedented findings, many of the data presented below are in line with published data, thereby assuring the validity of the experiments.

According to animal welfare guidelines, the minimal number of animals per group should be used to be able to detect differences between different groups. This was achieved in the study: In spite of the relative small group size (six animals per group), a large number of metabolites and clinical parameters that were regulated with statistical significance was observed, implying that the experimental conditions could be controlled well enough to assure reproducibility between individual animals.

While we cannot comment on all observed changes, selected marker classes that reflect major physiological pathways will be highlighted in the following sections.

### Hyperglycemia

**Fig 1:**
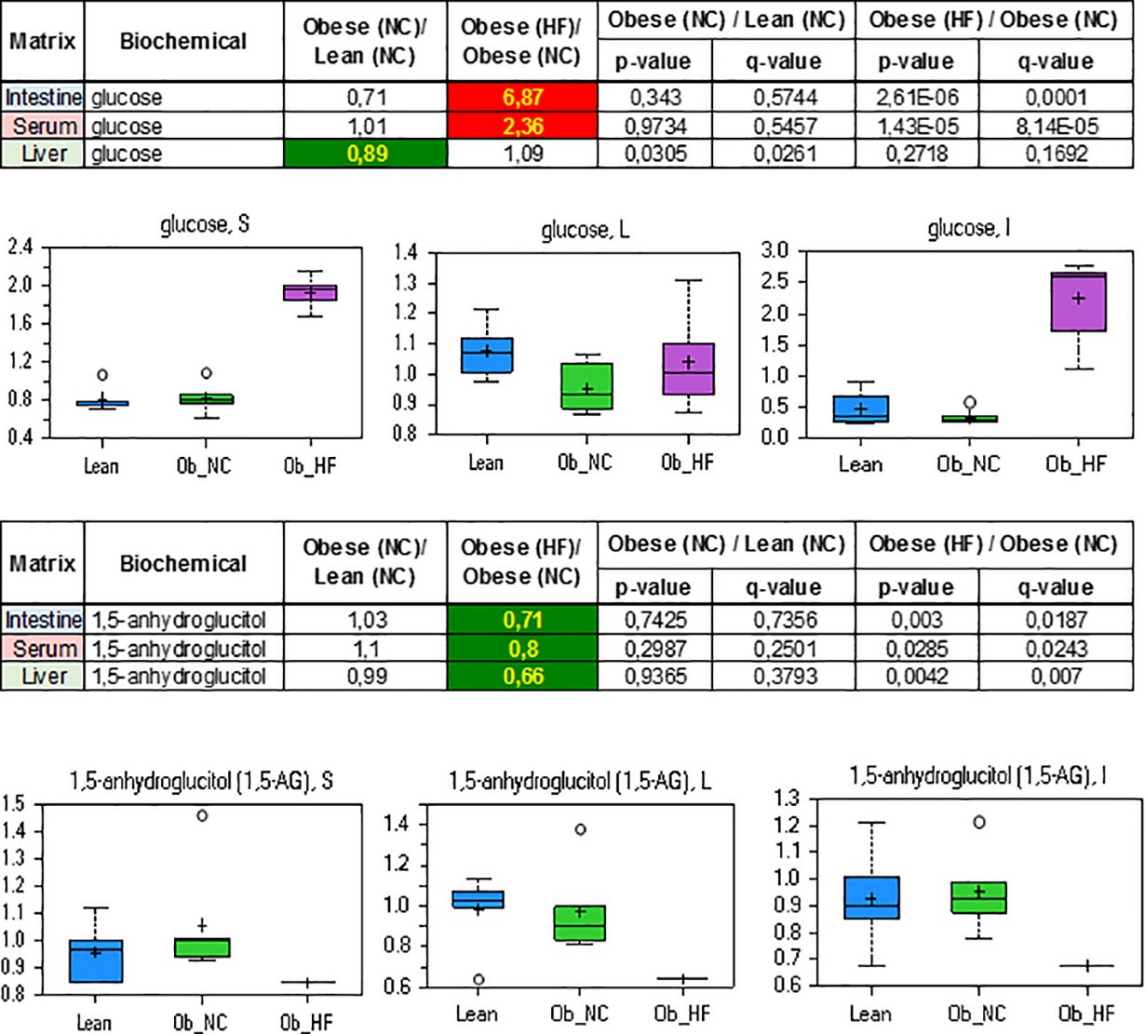
**Relative levels and concentration ratios of glucose (upper panel) and 1,5-anhydroglucitol (lower panel) in serum, liver and intestine**. The fold change for the matrices indicated is presented in the excerpt from the heat map table (green: indicates significant difference (p≤0.05) between the groups shown; red: indicates significant difference (p≤0.05) between the groups shown).

Dramatic increases in serum and intestinal levels of glucose were observed in the obese high fat diet fed ZDF rats consistent with hyperglycemia, a hallmark of type 2 diabetes (Fig 1). The hepatic levels of glucose were slightly increased, reflecting the constant effort by the liver to maintain glucose homeostasis.

Further indications that the obese ZDF rat on high fat diet had developed hyperglycemia were seen in the significantly decreased levels of 1,5-anhydroglucitol (1,5-AG) in serum, liver and intestine. 1,5-AG is derived mainly from food, is well absorbed in the intestinal tract, distributed widely to all organs and tissues, and eventually excreted in the urine in unchanged form when its level exceeds the renal threshold.[36–38] As the reabsorption of 1,5-AG in the renal tubules is competitively inhibited by glucosuria, increasing levels of glucosuria lead to lower amounts of 1,5-AG in the serum in humans.[36–38] The correlation between this reduction and the amount of glucose present in urine is so close that 1,5-AG represents a sensitive, real-time marker of glycemic control [36]. Indeed, lower levels of 1,5-AG were observed in serum, liver and intestine in obese diabetic ZDF rats, consistent with hyperglycemia (Fig 1).

**Fig 2:**
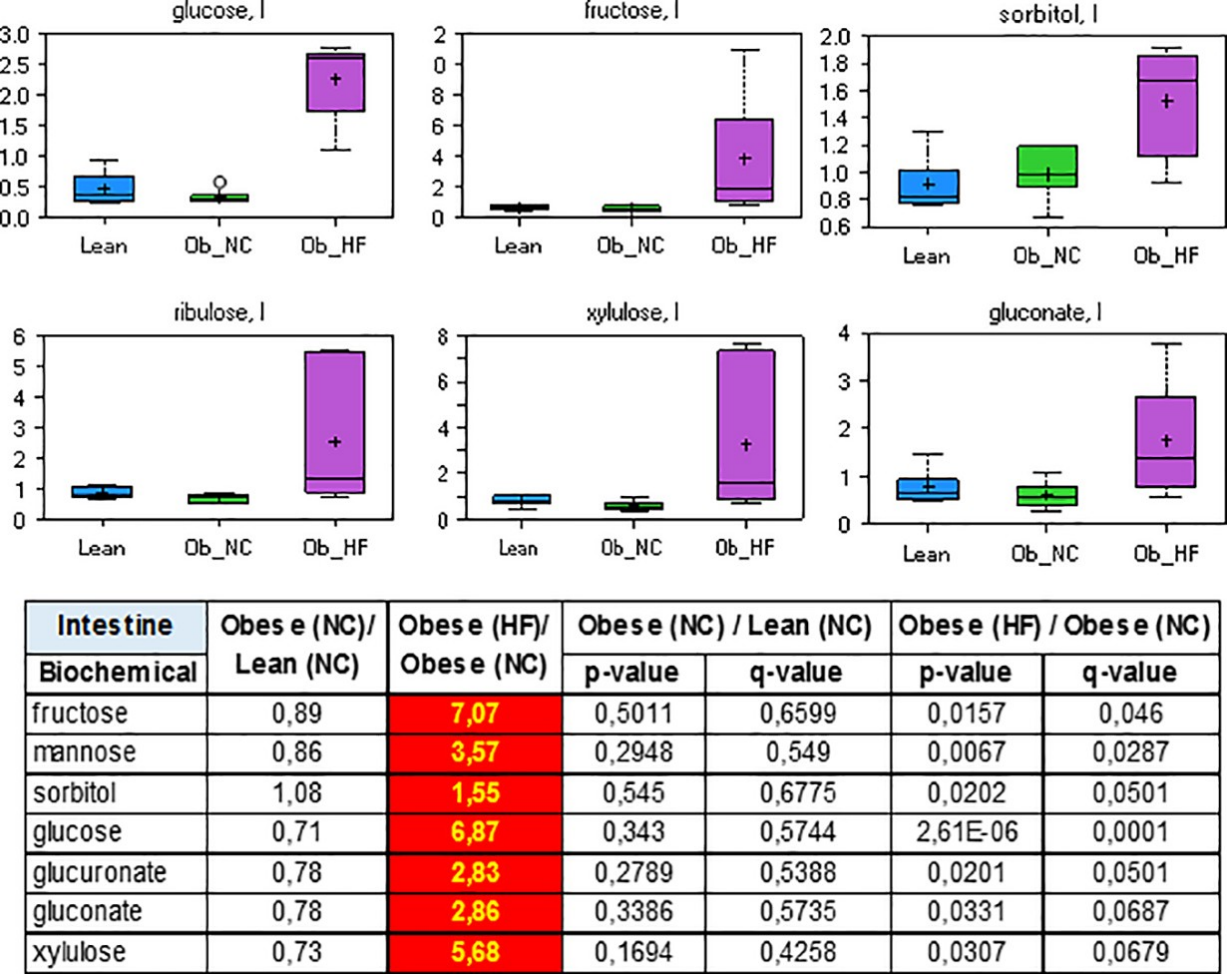
Relative levels and concentration ratios of saccharides in the intestine (red: Indicates significant difference (p≤0.05) between the groups shown).

In addition to elevated glucose, the concentrations of many other saccharides including fructose, sorbitol, ribulose, xylulose or gluconate were strongly increased in the intestine and the serum of diabetic ZDF rats (Fig 2 and S1 File, Tables B and F). Diabetes mellitus in rats is associated with enhanced intestinal absorption of glucose [39–41]. Several glucose transporters (GLUT), including GLUT2 and GLUT5, are also able to transport primary digestion products such as sugars and disaccharides. Thus, the observation is consistent with increased glucose uptake and may reflect the impaired ability of the Obese (HF) group to deal with elevated sugar levels.

### Lipids and ketone bodies

The abundance of free fatty acids showed marked differences between the three groups of animals (Tables 3–5). In serum and liver, ZDF rats (fa/fa) on normal chow had much higher levels of free fatty acids compared to (Fa/?) rats on normal chow. This concerned almost all fatty acids, irrespective of chain length and degree of saturation. The differences between ZDF rats (fa/fa) on normal chow and their diabetic counterpart on high fat diet were less pronounced and more subtle: While a further increase of serum fatty acid levels was observed in particular for uneven, long chain length species (C14-C19), the abundance of medium chain fatty acids (C6-C11) and of essential poly-unsaturated fatty acids decreased (Table 3). The abundance pattern in the intestine was different, in particular with respect to a decrease of fatty acids with medium chain length (C15-C19) in the obese group on normal chow compared to the lean control (Table 5).

**Fig 3:**
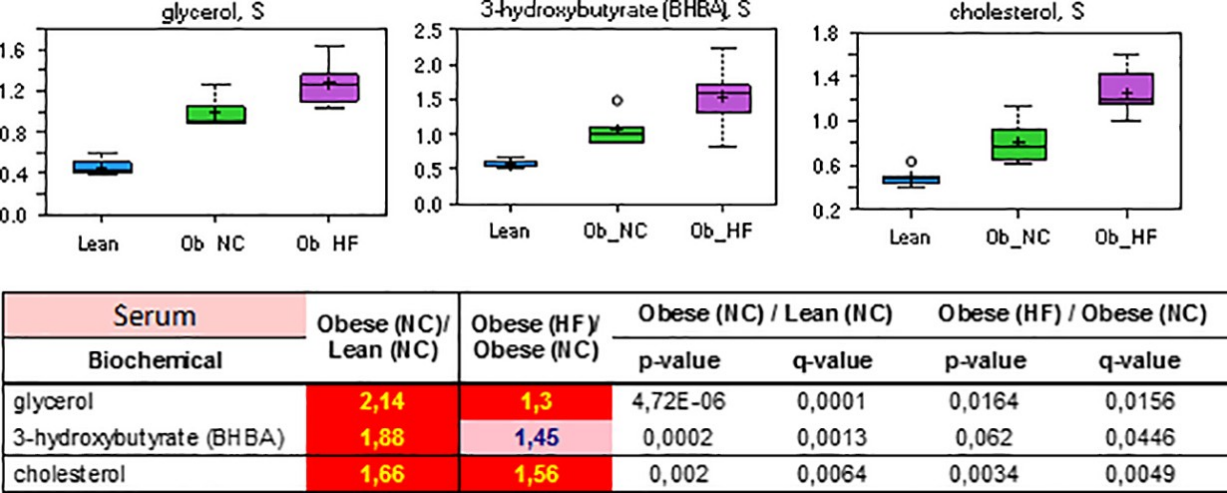
Relative levels and concentration ratios of the triacylglyceride degradation product glycerol, the fatty acid β-oxidation product 3-hydroxybutyrate, and cholesterol in serum (red: Indicates significant difference (p≤0.05) between the groups shown).

**Table 3:** Concentration ratios of fatty acids in the serum (the color code is defined in the materials and methods section).

Serum	Obese (NC)/ Lean (NC)	Obese (HF)/ Obese (NC)	Obese (NC) / Lean (NC)		Obese (HF) / Obese (NC)	
Biochemical			p-value	q-value	p-value	q-value
isocaproate	1,01	1	0,9941	0,5535	0,9456	0,3819
caproate (6:0)	1,16	**0,79**	0,2343	0,2116	0,0428	0,0341
heptanoate (7:0)	1,19	**0,54**	0,2936	0,2477	0,0022	0,0033
caprylate (8:0)	1,3	**0,54**	0,1454	0,1531	0,0038	0,0052
pelargonate (9:0)	**1,43**	**0,5**	0,0644	0,0844	0,0026	0,0038
caprate (10:0)	**1,45**	**0,55**	0,0247	0,0411	0,0017	0,003
undecanoate (11:0)	1,24	**0,63**	0,2084	0,1963	0,014	0,0138
laurate (12:0)	**1,25**	1	0,0324	0,0492	0,9725	0,3871
myristate (14:0)	**1,85**	1,3	0,0021	0,0066	0,1881	0,1084
myristoleate (14:1n5)	**1,79**	**1,75**	1,69E-06	9,69E-05	1,35E-06	1,17E-05
palmitate (16:0)	**1,55**	0,95	0,0006	0,0028	0,5429	0,2571
palmitoleate (16:1n7)	**2,55**	1,08	7,55E-06	0,0002	0,6978	0,3103
margarate (17:0)	1,09	**2,83**	0,4752	0,3503	0,0013	0,0024
10-heptadecenoate (17:1n7)	**1,26**	**2,95**	0,0119	0,0245	0,0002	0,0005
stearate (18:0)	**1,53**	**1,41**	0,0006	0,0027	0,0169	0,0159
oleate (18:1n9)	**1,61**	**1,44**	0,0002	0,0015	0,0282	0,0242
linoleate (18:2n6)	**0,7**	1,02	0,0006	0,0028	0,9304	0,3801
stearidonate (18:4n3)	**1,62**	**3,77**	0,0063	0,0149	2,50E-07	3,44E-06
nonadecanoate (19:0)	**0,7**	**2,34**	0,0327	0,0494	0,0101	0,011
10-nonadecenoate (19:1n9)	1,02	**4,3**	0,8073	0,5005	5,33E-05	0,0002
eicosenoate (20:1n9 or 11)	0,99	**1,51**	0,9913	0,5534	0,0527	0,0396
dihomo-linoleate (20:2n6)	**1,38**	1,22	0,0039	0,0112	0,3682	0,1873
mead acid (20:3n9)	**3,53**	1,18	1,14E-06	9,69E-05	0,1002	0,0658
dihomo-linolenate (20:3n3/n6)	**2,48**	**0,7**	0,0004	0,002	0,0575	0,0421
eicosapentaenoate (20:5n3)	**1,44**	0,9	0,0614	0,0815	0,4671	0,2284
arachidonate (20:4n6)	**1,41**	**0,83**	0,0034	0,01	0,0642	0,0458
adrenate (22:4n6)	**1,98**	**0,61**	0,0001	0,0011	0,0068	0,0081
docosapentaenoate (22:5n3)	**1,98**	**0,48**	0,0011	0,0041	0,0051	0,0066
docosapentaenoate (22:5n6)	**1,96**	**0,66**	0,0011	0,0041	0,0042	0,0056
docosahexaenoate (22:6n3)	**1,84**	**0,76**	1,33E-05	0,0003	0,0519	0,0394
1,2-dipalmitoylglycerol	**2,72**	**1,41**	0,001	0,0039	0,0952	0,0635
1,3-dipalmitoylglycerol	**3,63**	1,06	0,0059	0,0142	0,7997	0,3423

**Table 4:** Concentration ratios of fatty acids in the liver (the color code is defined in the materials and methods section).

Liver	Obese (NC)/ Lean (NC)	Obese (HF)/ Obese (NC)	Obese (NC) / Lean (NC)		Obese (HF) / Obese (NC)	
Biochemical			p-value	q-value	p-value	q-value
caproate (6:0)	1	1,25	0,9218	0,3768	0,6685	0,3253
caprylate (8:0)	0,88	**0,75**	0,7964	0,3425	0,0488	0,0471
pelargonate (9:0)	0,88	**0,85**	0,172	0,0972	0,0583	0,0538
caprate (10:0)	0,89	0,89	0,188	0,1044	0,2272	0,1473
laurate (12:0)	**0,9**	0,99	0,0599	0,0431	0,8785	0,3949
myristate (14:0)	**2,09**	**0,75**	1,29E-05	0,0001	0,0178	0,0224
myristoleate (14:1n5)	**1,29**	**0,78**	0,0003	0,0008	0,0058	0,0087
pentadecanoate (15:0)	**1,37**	**1,53**	0,0047	0,0057	0,0002	0,0007
palmitate (16:0)	**1,8**	0,93	0,0002	0,0006	0,4933	0,2632
palmitoleate (16:1n7)	**2,56**	**0,74**	1,28E-05	0,0001	0,0241	0,0282
margarate (17:0)	**1,51**	**2,36**	0,0078	0,0086	5,19E-05	0,0003
10-heptadecenoate (17:1n7)	**1,66**	**2,04**	7,18E-06	9,51E-05	2,30E-05	0,0002
stearate (18:0)	**1,61**	1,02	4,98E-05	0,0003	0,7562	0,3613
oleate (18:1n9)	**3,62**	0,99	6,05E-07	1,61E-05	0,9529	0,4113
linoleate (18:2n6)	**1,23**	1,01	0,0137	0,0135	0,844	0,384
linolenate (18:3n3/6)	**1,87**	**0,79**	0,0007	0,0013	0,0895	0,0762
stearidonate (18:4n3)	**3,09**	**0,63**	1,88E-05	0,0001	0,0125	0,0165
nonadecanoate (19:0)	**0,8**	**1,49**	0,0986	0,0632	0,0047	0,0074
10-nonadecenoate (19:1n9)	**2,15**	**2,92**	9,95E-05	0,0004	6,88E-06	0,0001
eicosenoate (20:1n9/11)	**2,18**	**1,49**	0,0007	0,0013	0,0319	0,0343
dihomo-linoleate (20:2n6)	**2,01**	1,26	0,0004	0,0009	0,1047	0,0842
mead acid (20:3n9)	**4,12**	**1,48**	2,14E-05	0,0002	0,0876	0,0752
dihomo-linolenate (20:3n3/n6)	**2,5**	1,03	8,12E-06	9,51E-05	0,9032	0,3993
arachidonate (20:4n6)	**1,38**	0,91	0,0013	0,0021	0,2966	0,1792
eicosapentaenoate (20:5n3)	**2,03**	**0,77**	6,25E-05	0,0003	0,0879	0,0752
docosadienoate (22:2n6)	**2,58**	0,91	0,0005	0,0011	0,6531	0,3219
docosatrienoate (22:3n3)	**8,74**	**1,56**	1,88E-06	3,50E-05	0,0529	0,0501
adrenate (22:4n6)	**2,3**	0,85	5,25E-05	0,0003	0,2742	0,1695
docosahexaenoate (22:6n3)	**1,84**	**1,14**	9,00E-06	9,78E-05	0,0685	0,0617

**Table 5:** Concentration ratios of fatty acids in the intestine (the color code is defined in the materials and methods section).

Intestine	Obese (NC)/ Lean (NC)	Obese (HF)/ Obese (NC)	Obese (NC) / Lean (NC)		Obese (HF) / Obese (NC)	
Biochemical			p-value	q-value	p-value	q-value
caproate (6:0)	1,07	0,79	0,8632	0,7613	0,3392	0,2633
heptanoate (7:0)	1,24	0,63	0,9762	0,7906	0,4415	0,3039
caprylate (8:0)	1,04	0,86	0,7767	0,7505	0,166	0,1781
pelargonate (9:0)	1,01	0,89	0,9782	0,7906	0,246	0,2173
caprate (10:0)	1,05	1,05	0,8109	0,7505	0,9242	0,4598
laurate (12:0)	1,05	**4,07**	0,4516	0,6246	0,0129	0,0425
myristate (14:0)	0,82	1,75	0,2205	0,4979	0,1263	0,1474
myristoleate (14:1n5)	0,81	**2,42**	0,3845	0,5855	0,0933	0,1252
pentadecanoate (15:0)	**0,47**	0,99	1,02E-05	0,0048	0,8419	0,4402
palmitate (16:0)	0,97	0,91	0,6806	0,7214	0,2274	0,2099
palmitoleate (16:1n7)	1,18	1,1	0,3069	0,551	0,8731	0,4476
margarate (17:0)	**0,64**	**2,04**	0,0054	0,086	0,0004	0,0045
10-heptadecenoate (17:1n7)	**0,8**	**2,69**	0,0977	0,3149	0,0003	0,0038
stearate (18:0)	**0,87**	**1,18**	0,0458	0,2296	0,0093	0,0349
oleate (18:1n9)	**1,19**	**1,42**	0,0707	0,2711	0,0043	0,0224
linoleate (18:2n6)	**0,67**	0,9	0,0024	0,0558	0,3509	0,2663
linolenate (18:3n3/n6)]	**0,68**	1	0,0042	0,0804	0,7719	0,416
nonadecanoate (19:0)	**0,45**	**1,26**	0,0005	0,0269	0,0963	0,1274
10-nonadecenoate (19:1n9)	**0,72**	**2,79**	0,0354	0,2033	3,82E-05	0,0009
eicosenoate (20:1n9 or 11)	1,18	1,06	0,4437	0,6229	0,7082	0,3925
dihomo-linoleate (20:2n6)	0,83	0,83	0,3068	0,551	0,3344	0,263
mead acid (20:3n9)	**2,43**	1,64	0,0003	0,0269	0,3392	0,2633
dihomo-linolenate (20:3n3/n6)	1,3	0,85	0,1341	0,3775	0,3305	0,2614
arachidonate (20:4n6)	1,23	1,19	0,2026	0,4807	0,1525	0,1703
eicosapentaenoate (20:5n3)	**1,56**	1,15	0,0348	0,2033	0,3429	0,2653
docosadienoate (22:2n6)	0,96	0,71	0,9149	0,7744	0,1993	0,1976
docosatrienoate (22:3n3)	0,97	**0,27**	0,9071	0,7744	0,0006	0,0056
adrenate (22:4n6)	1,27	0,78	0,2462	0,516	0,2089	0,2013
docosapentaenoate (22:5n3)	**1,48**	0,94	0,0685	0,2646	0,4564	0,3076
docosapentaenoate (22:5n6)	**1,8**	1,16	0,0143	0,1362	0,7536	0,4105
docosahexaenoate (22:6n3)	1,21	1,14	0,1634	0,4195	0,2082	0,2013

The elevated levels of fatty acids in serum and the liver are consistent with hyperlipidemia (see Table 1). Insulin promotes lipogenesis, triglyceride synthesis and accumulation in adipose tissue through its coordinated activation of lipoprotein lipase and inhibition of hormone-sensitive lipase in rodents and in humans. As a result of these actions, free fatty acids are removed from the circulation and re-esterified as intracellular triglycerides. However, the ability of insulin to promote this process is impaired in an insulin resistant state, and increased lipolysis from an expanded adipose tissue (increased fat mass) in obese animals further contributes to the rise of circulating levels of fatty acids. This interpretation is also supported by the finding that the serum level of free glycerol, the by-product of lipolysis, was increased in the order lean (NC) < obese (NC) < obese (HF) (Fig 3). The same order of abundances was observed for total cholesterol in serum and the liver as a marker of dyslipidemia. Dietary effects may be responsible for the decreased levels of essential poly-unsaturated fatty and of medium chain fatty acids (C6-C11). The latter species may additionally be altered by microbiota metabolism, and by an increase of de novo fatty acid synthesis. In that case, the increased synthesis of uneven, long chain length species (C14-C19) under substrate limitation condition may have induced the depletion of medium chain fatty acid precursors.

While acetoacetate could not be reliably detected, increased levels of ketone body 3-hydroxybutyrate were observed in serum of obese diabetic ZDF rats, consistent with an increased β-oxidation activity (Fig 3). This finding is in line with previous studies that report an induction of β-oxidation genes in humans or rodents upon feeding of a high fat diet, or in cultures of muscle cells exposed to high levels of fatty acids.[42] As glucose disposal becomes increasingly inefficient, cells become more dependent on fatty acids as a fuel source via β-oxidation. In this process, a small fraction of acetyl-CoA generated from β-oxidation of fatty acids is converted to the ketone bodies acetoacetate and 3-hydroxybutyrate in the liver.

Acylcarnitines have been associated with insulin resistance, reflecting incomplete fatty acid oxidation processes.[43,44] Compared with the lean control, an increase of most carnitine species derived from long and short chain fatty acids as well as amino acid metabolism was observed in the sera of the obese group (S1 File, Table B). This finding is consistent with the higher lipid levels found in these animals that demand a largely increased β-oxidation (and subsequent TCA cycle) capacity. However, in particular the levels of short- and medium-chain acylcarnitines in diabetic rats under a high fat diet were reduced compared to the obese (NC) group. This mirrors the reduction of the corresponding free fatty acids in serum (Table 3). As a carnitine-independent uptake and intramitochondrial activation to acyl-CoA thioesters has been reported for short- and medium-chain acylcarnitines, we assume that the reduced levels do not indicate a change in b-oxidation, but probably reflects an altered dietary content.

### Bile acids

In serum, intestine and liver, significant alterations in levels of various bile acids were observed. In the obese group receiving normal chow, most unconjugated and conjugated bile acid levels decreased compared to the lean control groups in serum, liver and intestine (Tables 6–8). In the diabetic group that received a high fat diet, in particular cholate and deoxycholate species in the intestine were significantly upregulated compared to the obese group receiving a normal chow. Bile acids are synthesized from cholesterol in the liver and form bile in order to facilitate dietary lipid absorption. The elevation in the high fat diet group may be a direct response to the high fat diet to facilitate uptake. It is also possible that elevated bile acids are associated with attempts to reduce cholesterol by converting it to bile acids. Recently, it has become clear that these agents can function as hormones in humans in the regulation of various metabolic processes, including glycemic control,[45–47] e.g. via stimulation of the farnesoid X receptor (FXR) and/or the TGR5 receptor.[45,48] The global reduction of bile acid levels in the fa/fa genotype under normal chow compared to the Fa/? lean control was unexpected, but may reflect the leptin-mediated regulation of bile acid synthesis.[49] In fact, a recent study also reported elevated bile acid levels in ZDF rats upon improved metabolic conditions following a Roux-en-Y gastric bypass surgery.[50] On the other hand, a study that compared biliary lipids in fa/fa vs. Fa/? ZDF rats found rather constant levels of bile acid species in the bile fistulae.[51]

**Table 6:** Concentration ratios of bile acids in the serum (the color code is defined in the materials and methods section).

Serum	Obese (NC)/ Lean (NC)	Obese (HF)/ Obese (NC)	Obese (NC) / Lean (NC)		Obese (HF) / Obese (NC)	
Biochemical			p-value	q-value	p-value	q-value
glycocholate	**0,53**	**2,13**	0,0291	0,0451	0,0186	0,0171
taurocholate	**0,21**	**4,5**	3,23E-05	0,0006	0,0124	0,0127
chenodeoxycholate	1,18	**0,07**	0,7684	0,4853	0,0014	0,0024
taurochenodeoxycholate	**0,15**	0,93	2,47E-06	0,0001	0,4284	0,212
deoxycholate	0,64	1,08	0,1903	0,1873	0,3857	0,1932
taurodeoxycholate	**0,16**	**4,27**	3,56E-05	0,0006	0,0145	0,0142
beta-muricholate	1,06	**0,32**	0,9302	0,5362	0,0797	0,0549
alpha-muricholate	1,21	**0,11**	0,8832	0,5276	0,0189	0,0172

**Table 7:** Concentration ratios of bile acids in the liver (the color code is defined in the materials and methods section).

Liver	Obese (NC)/ Lean (NC)	Obese (HF)/ Obese (NC)	Obese (NC) / Lean (NC)		Obese (HF) / Obese (NC)	
Biochemical			p-value	q-value	p-value	q-value
glycocholate	0,83	1,23	0,3328	0,1696	0,3856	0,2192
taurocholate	**0,68**	1,1	0,0307	0,0261	0,7404	0,3556
taurochenodeoxycholate	**0,49**	**0,24**	0,0005	0,0011	9,91E-06	0,0001
taurodeoxycholate	**0,39**	1,55	0,0002	0,0007	0,2093	0,138
glycodeoxycholate	0,72	0,96	0,608	0,2765	0,8143	0,3768
glycochenodeoxycholate	0,72	**0,2**	0,5044	0,2362	0,0005	0,0014
taurolithocholate	**0,24**	0,65	0,0004	0,001	0,2377	0,1522
tauroursodeoxycholate	0,72	**0,47**	0,1887	0,1044	0,0075	0,0107

**Table 8:** Concentration ratios of bile acids in the intestine (the color code is defined in the materials and methods section).

Intestine	Obese (NC)/ Lean (NC)	Obese (HF)/ Obese (NC)	Obese (NC) / Lean (NC)		Obese (HF) / Obese (NC)	
Biochemical			p-value	q-value	p-value	q-value
glycocholate	0,54	**4,07**	0,1418	0,3904	0,0084	0,0343
taurocholate	**0,48**	**3,87**	0,0633	0,2628	0,0085	0,0345
chenodeoxycholate	0,32	**0,3**	0,3786	0,5855	0,0332	0,0687
taurochenodeoxycholate	**0,27**	1,53	0,0063	0,0869	0,652	0,3797
taurodeoxycholate	**0,15**	**7,67**	0,0062	0,0869	0,0054	0,0263
glycochenodeoxycholate	**0,27**	1,1	0,0487	0,2372	0,5878	0,3583
alpha-muricholate	0,6	**0,26**	0,9919	0,7906	0,041	0,0754
taurocholenate sulfate*	**0,47**	**3,05**	0,0637	0,2628	0,0141	0,0432
glycoursodeoxycholate	0,47	**1,98**	0,2291	0,4995	0,0041	0,0224
tauroursodeoxycholate	**0,47**	**2,23**	0,0511	0,2436	0,0893	0,1227

### Amino acid utilization

Almost all proteinogenic amino acids were of significantly lower abundance in the liver of obese diabetic rats compared to the obese control under normal chow. The degree of regulation was moderate, i.e. less than 2-fold in all cases. It is noteworthy that in the serum, two out of the three branched chained amino acids (BCAA), isoleucine and valine, were elevated (Tables 9 and 10).

**Table 9:** Concentration ratios of amino acid levels in the serum (the color code is defined in the materials and methods section).

Serum	Obese (NC)/ Lean (NC)	Obese (HF)/ Obese (NC)	Obese (NC) / Lean (NC)		Obese (HF) / Obese (NC)	
Biochemical			p-value	q-value	p-value	q-value
glycine	**0,59**	1,2	0,0014	0,0053	0,2532	0,1373
serine	0,89	0,96	0,3793	0,3021	0,7608	0,3299
threonine	**1,4**	**0,67**	0,0879	0,1085	0,0399	0,0323
aspartate	**1,68**	0,82	0,0199	0,0348	0,3715	0,1883
asparagine	**0,63**	**1,31**	7,72E-05	0,0008	0,0035	0,0049
alanine	1,06	**0,75**	0,5072	0,3644	0,0907	0,0615
glutamate	**1,4**	0,96	0,0785	0,1	0,7895	0,3396
glutamine	**0,81**	0,86	0,0923	0,1115	0,3088	0,162
histidine	0,99	**0,86**	0,928	0,5362	0,0455	0,0359
lysine	1,01	**0,44**	0,9106	0,5349	0,0042	0,0056
phenylalanine	**1,24**	**0,86**	0,0062	0,0149	0,0106	0,0113
tyrosine	1,12	1,05	0,2003	0,1931	0,92	0,3771
tryptophan	0,93	**0,81**	0,1961	0,1915	0,0262	0,0227
isoleucine	1,09	**1,44**	0,1395	0,1481	0,0006	0,0013
leucine	1,11	**0,83**	0,2637	0,2312	0,0575	0,0421
valine	1,11	**1,12**	0,1999	0,1931	0,0934	0,0626
cysteine	0,98	**0,72**	0,8057	0,5005	0,0172	0,0161
methionine	1,01	**0,64**	0,9752	0,5457	8,70E-05	0,0003
arginine	0,99	**0,74**	0,7699	0,4853	0,0967	0,064
proline	1,02	**0,72**	0,8363	0,5076	0,0008	0,0016

**Table 10:** Concentration ratios of amino acid levels in the liver (the color code is defined in the materials and methods section).

Liver	Obese (NC)/ Lean (NC)	Obese (HF)/ Obese (NC)	Obese (NC) / Lean (NC)		Obese (HF) / Obese (NC)	
Biochemical			p-value	q-value	p-value	q-value
glycine	0,9	0,9	0,1073	0,0671	0,1975	0,1326
serine	1,08	**0,64**	0,3104	0,1596	0,0012	0,0027
threonine	**1,86**	**0,5**	4,80E-05	0,0003	0,0023	0,0043
aspartate	**1,83**	**0,68**	0,0026	0,0035	0,0397	0,0406
asparagine	0,87	**0,58**	0,5972	0,2738	0,0334	0,0353
alanine	**1,11**	0,9	0,0399	0,032	0,1875	0,1264
glutamate	1,07	1,08	0,1463	0,0868	0,4994	0,265
glutamine	**0,85**	**0,64**	0,0019	0,0027	0,0003	0,0011
histidine	**1,07**	**0,79**	0,0984	0,0632	0,002	0,004
lysine	**0,95**	**0,82**	0,048	0,0372	0,0003	0,0011
phenylalanine	0,99	**0,59**	0,8755	0,3639	2,81E-05	0,0002
tyrosine	1,05	**0,67**	0,5575	0,257	0,0006	0,0016
tryptophan	0,91	**0,59**	0,1309	0,0798	1,09E-05	0,0001
isoleucine	1,01	**0,63**	0,9972	0,3956	0,0003	0,001
leucine	0,96	**0,61**	0,4625	0,2201	1,05E-05	0,0001
valine	**0,89**	**0,69**	0,0386	0,0314	4,26E-05	0,0003
cysteine	1,05	0,85	0,6267	0,2817	0,1304	0,0985
methionine	**0,9**	**0,68**	0,0388	0,0314	1,99E-05	0,0002
arginine	0,97	0,65	0,5475	0,2537	0,1309	0,0985
proline	1,1	**0,71**	0,1347	0,0813	1,04E-05	0,0001

Glucogenic amino acids are degraded to one of the glucose precursors such as pyruvate, α-ketoglutarate, succinyl-CoA, fumarate or oxaloacetate. Relative to normal controls, levels of glucogenic amino acids have been shown to be reduced in both human and non-human models of diabetes. This is considered to be a consequence of impaired glucose uptake in insulin-resistant cells.[52,53] Resultant stimulation of hepatic gluconeogenesis consumes these glucogenic amino acids in order to deliver glucose precursors. Ketogenic amino acids are degraded to either acetyl-CoA or acetoacetate and therefore can be converted to fatty acids or ketone bodies. The observed decreased levels of ketogenic amino acids are consistent with their degradation and the concomitant elevated levels of 3-hydroxybutyrate and fatty acids (see above).

Increased levels of BCAAs in the blood have been suggested to be a contributor to the development of obesity-associated insulin resistance,[42,54] and as a predictor for type 2 diabetes in humans.[55] Also She *et al*. reported elevated levels of BCAA’s in the plasma of male obese ZDF rats, but found comparable concentrations in the liver.[56] Kwak *et al*. recently published a focused study of sulfur amino acid metabolism in ZDF rats.[57] In line with the findings of this study, they observed a decrease of cysteine and methionine in the plasma and liver of obese ZDF rats.

### Oxidative stress–Antioxidant response system

Oxidative stress plays an important role in the pathogenesis and the complications of diabetes.[58] Chronically high levels of free radicals and insufficiently robust antioxidant defense mechanisms can lead to damage of cellular organelles and enzymes, increased lipid peroxidation and development of insulin resistance, thereby further promoting the development of complications of diabetes mellitus.

**Fig 4:**
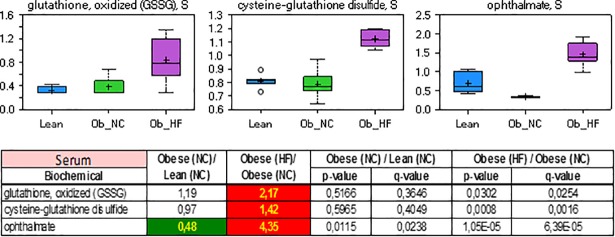
Relative levels and concentration ratios of oxidized glutathione, cysteine-glutathione and ophthalmate in serum (green: Indicates significant difference (p≤0.05) between the groups shown; red: Indicates significant difference (p≤0.05) between the groups shown).

In this study, increased levels of oxidized glutathione (GSSG), cysteine-glutathione disulfide and ophthalmate were observed in obese diabetic rats (Fig 4). The tripeptide antioxidant glutathione is a major part of the antioxidant response system by providing reducing equivalents to glutathione peroxidase. The elevated levels of GSSG indicate that its recycling to GSH via glutathione reductase was limited. The direct quantification of GSH in serum and tissue has not been feasible in this and other studies due to its instability. Ophthalmate, a structural analogue of GSH that has a 2-aminobutyrate in the active site instead of cysteine, has been proposed as a surrogate marker of hepatic GSH depletion in humans.[59] Because it is synthesized under cysteine-limited conditions at the expense of glutathione, its level was reported to be inversely correlated to GSH in the mouse liver[60] as well as in mouse embryo fibroblasts.[61] Thus, the elevated serum and liver levels of ophthalmate observed in this study are consistent with oxidative stress in diabetic rats of the high fat diet group.

### Pioglitazone-induced effects

Pioglitazone predominantly acts on PPARγ and to a lesser extent PPARα. It thereby modulates the transcription of insulin-sensitive genes involved in the control of glucose and lipid metabolism in muscle, adipose tissue, and liver. Pioglitazone decreases insulin resistance in the periphery and in the liver, resulting in increased insulin-dependent glucose disposal and decreased hepatic glucose output. Not surprisingly, these actions have a pronounced effect of the metabolome of the matrices investigated in this study. For example, of the 443 biochemicals detected in the liver, 114 were upregulated by 50% or higher, and 41 were downregulated to this extent compared to the levels observed in untreated obese rats under high fat diet. The detection of pioglitazone itself as well as its metabolite hydroxypioglitazone,[62] positive only in the drug-treated group, confirmed that an exposure to the drug has been achieved by the administration scheme (S1 File, Tables A-F).

In order to probe whether pioglitazone is able to reverse the pathogenesis of diabetes in the animal model on the metabolome level, metabolite changes are depicted in relation to the effects induced by the high fat diet. The observed changes of several clinical biomarkers listed in Table 1, e.g. adiponectin, liver weight, body weight, or triglycerides, are the consequence of a chronic treatment and do not represent acute effects. Thus, a chronic treatment effect definitely exists, and we do not expect pronounced metabolome-wide acute effects within one hour after treatment. A rapidly regulated class of metabolites are free fatty acids (t_1/2_: 3–5 min). In particular, high levels of free fatty acids can be reduced by insulin within minutes. However, the reduced free fatty acids in pioglitazone-treated rats appeared in the presence of reduced serum insulin levels. Therefore, we conclude that the reduced free fatty acid levels in pioglitazone-treated rats are very likely a chronic treatment effect of the drug. However, we cannot exclude that some metabolite changes may represent an acute response to pioglitazone. This represents a limitation of the current study.

Pioglitazone indeed induced pronounced changes to the metabolome in various metabolic superpathways, as outlined below.

### Glucose handling

**Fig 5:**
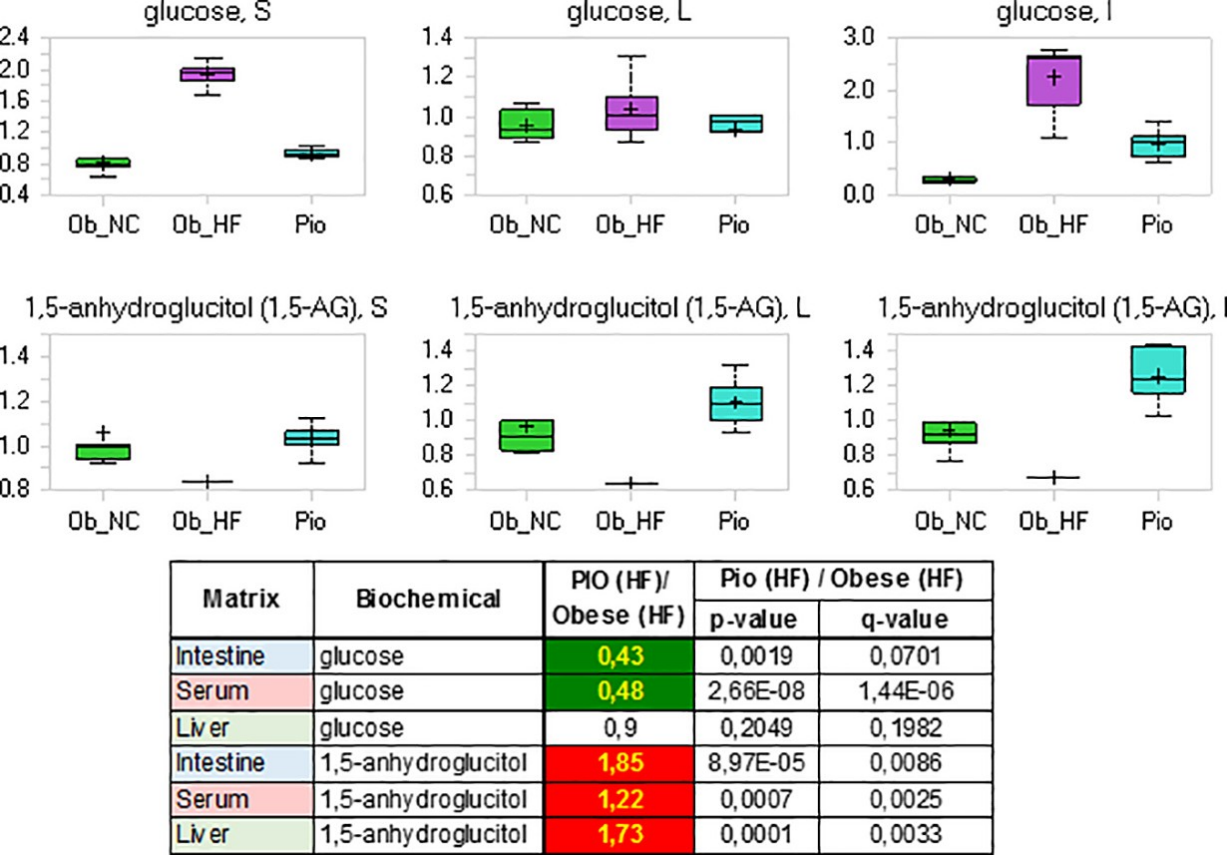
Relative levels and concentration ratios of glucose and 1,5-anhydroglucitol in serum, intestine and liver (green: Indicates significant difference (p≤0.05) between the groups shown; red: Indicates significant difference (p≤0.05) between the groups shown).

A primary goal for any effective anti-diabetic therapy is to reestablish glycemic control. As shown in Fig 5, pioglitazone was able to lower glucose in both serum and intestine, consistent with the expected effect of the drug. Glucose levels in the liver were unaffected, but remained on the same level as those of non-diabetic control animals (see above). Further indications that pioglitazone improved glycemic control were provided by significant changes in serum, intestine and liver levels of 1,5-AG, that were elevated to the levels found in healthy, non-diabetic animals (Fig 5).

### Lipids and ketone bodies

As described above, the diabetic ZDF rat experienced dyslipidemia with a distinct signature of free fatty acids in the serum, featured by an increase in saturated or monounsaturated species of long chain length (MUFAs) and a concomitant decrease of the abundance of medium chain fatty acids (C6-C11) and of essential poly-unsaturated fatty acids (PUFAs, C20-C22). Pioglitazone treatment led to a reversal of this profile with respect to the first two categories; in contrast, the abundance of PUFAs was further decreased upon treatment (Table 11). In sum, this led to a (non-significant, p = 0.06) decrease of NEFAs (Table 11). A similar pattern was observed in liver samples of this study (Table 12), while little effects were found in the intestine.

**Table 11:** Concentration ratios of fatty acids in the serum (the color code is defined in the materials and methods section).

Serum	Obese (NC)/ Lean (NC)	Pio (HF)/ Obese (HF)	Obese (NC) / Lean (NC)		Pio (HF) / Obese (HF)	
Biochemical			p-value	q-value	p-value	q-value
isocaproate	1	**0,53**	0,9456	0,3819	0,0011	0,0032
caproate (6:0)	**0,79**	**1,35**	0,0428	0,0341	0,0883	0,0751
heptanoate (7:0)	**0,54**	**1,85**	0,0022	0,0033	0,01	0,016
caprylate (8:0)	**0,54**	**2,1**	0,0038	0,0052	0,0021	0,0053
pelargonate (9:0)	**0,5**	**2,37**	0,0026	0,0038	0,0013	0,0037
caprate (10:0)	**0,55**	**2,34**	0,0017	0,003	0,0011	0,0032
undecanoate (11:0)	**0,63**	**2,21**	0,014	0,0138	0,005	0,0095
laurate (12:0)	1	**2,66**	0,9725	0,3871	0,0016	0,0045
myristoleate (14:1n5)	**1,75**	**2,11**	1,35E-06	1,17E-05	0,0412	0,0439
palmitate (16:0)	0,95	0,85	0,5429	0,2571	0,2087	0,1451
palmitoleate (16:1n7)	1,08	1,19	0,6978	0,3103	0,6785	0,3194
margarate (17:0)	**2,83**	**0,58**	0,0013	0,0024	0,0455	0,0466
10-heptadecenoate (17:1n7)	**2,95**	0,96	0,0002	0,0005	0,6816	0,3199
stearate (18:0)	**1,41**	**0,6**	0,0169	0,0159	0,0034	0,0078
oleate (18:1n9)	**1,44**	**0,67**	0,0282	0,0242	0,0426	0,0442
stearidonate (18:4n3)	**3,77**	**0,43**	2,50E-07	3,44E-06	3,54E-05	0,0004
nonadecanoate (19:0)	**2,34**	**0,5**	0,0101	0,011	0,0266	0,0309
10-nonadecenoate (19:1n9)	**4,3**	**0,46**	5,33E-05	0,0002	0,0064	0,0113
eicosenoate (20:1n9/n11)	**1,51**	**0,35**	0,0527	0,0396	0,0003	0,0014
dihomo-linoleate (20:2n6)	1,22	**0,49**	0,3682	0,1873	0,006	0,0107
mead acid (20:3n9)	1,18	**0,79**	0,1002	0,0658	0,0726	0,0664
dihomo-linolenate (20:3n3/n6)	**0,7**	0,87	0,0575	0,0421	0,248	0,1616
arachidonate (20:4n6)	**0,83**	**0,73**	0,0642	0,0458	0,0361	0,0394
adrenate (22:4n6)	**0,61**	**0,4**	0,0068	0,0081	0,0005	0,0019
docosapentaenoate (22:5n3)	**0,48**	1,24	0,0051	0,0066	0,5138	0,2682
docosapentaenoate (22:5n6)	**0,66**	**0,27**	0,0042	0,0056	1,99E-07	8,11E-06
docosahexaenoate (22:6n3)	**0,76**	**0,46**	0,0519	0,0394	0,0005	0,0021

**Table 12:** Concentration ratios of fatty acids in the liver (the color code is defined in the materials and methods section).

Liver	Obese (NC)/ Lean (NC)	Pio (HF)/ Obese (HF)	Obese (NC) / Lean (NC)		Pio (HF) / Obese (HF)	
Biochemical			p-value	q-value	p-value	q-value
caproate (6:0)	1,25	1,16	0,6685	0,3253	0,7538	0,3973
caprylate (8:0)	**0,75**	**1,45**	0,0488	0,0471	0,0299	0,0561
pelargonate (9:0)	**0,85**	1,24	0,0583	0,0538	0,1379	0,1518
caprate (10:0)	0,89	**1,25**	0,2272	0,1473	0,0666	0,0926
laurate (12:0)	0,99	**2,09**	0,8785	0,3949	4,07E-05	0,0016
myristate (14:0)	**0,75**	**1,67**	0,0178	0,0224	0,0003	0,0044
myristoleate (14:1n5)	**0,78**	**2,16**	0,0058	0,0087	3,09E-06	0,0005
pentadecanoate (15:0)	**1,53**	**1,48**	0,0002	0,0007	0,0217	0,0452
palmitate (16:0)	0,93	1,08	0,4933	0,2632	0,2937	0,2398
palmitoleate (16:1n7)	**0,74**	**1,58**	0,0241	0,0282	0,0029	0,0128
margarate (17:0)	**2,36**	0,91	5,19E-05	0,0003	0,3297	0,2549
10-heptadecenoate (17:1n7)	**2,04**	**1,5**	2,30E-05	0,0002	0,0032	0,0137
stearate (18:0)	1,02	0,88	0,7562	0,3613	0,1579	0,1647
oleate (18:1n9)	0,99	1,04	0,9529	0,4113	0,7323	0,3931
linolenate (18:3n3/n6)	**0,79**	0,88	0,0895	0,0762	0,2414	0,2121
stearidonate (18:4n3)	**0,63**	1,25	0,0125	0,0165	0,4316	0,2941
nonadecanoate (19:0)	**1,49**	**0,74**	0,0047	0,0074	0,032	0,0583
10-nonadecenoate (19:1n9)	**2,92**	0,91	6,88E-06	0,0001	0,4596	0,3015
eicosenoate (20:1n9/n11)	**1,49**	**0,57**	0,0319	0,0343	0,0118	0,0289
dihomo-linoleate (20:2n6)	1,26	**0,56**	0,1047	0,0842	0,0069	0,0215
mead acid (20:3n9)	**1,48**	**0,49**	0,0876	0,0752	0,036	0,0638
dihomo-linolenate (20:3n3/n6)	1,03	**0,72**	0,9032	0,3993	0,0632	0,0904
arachidonate (20:4n6)	0,91	**0,76**	0,2966	0,1792	0,0111	0,028
eicosapentaenoate (20:5n3)	**0,77**	0,86	0,0879	0,0752	0,3755	0,2731
docosatrienoate (22:3n3)	**1,56**	**0,42**	0,0529	0,0501	0,007	0,0215
adrenate (22:4n6)	0,85	**0,32**	0,2742	0,1695	0,0017	0,0098
docosahexaenoate 22:6n3)	**1,14**	**0,58**	0,0685	0,0617	0,0002	0,0033

Interestingly, Sato *et al*. also reported the fatty acid composition from insulin-resistant, high fat-fed rats that were treated with pioglitazone in a recently published study.[26] The authors investigated adipocytes isolated at study end, but measured the composition of the triglyceride fraction following hydrolysis, in contrast to our study that detected free fatty acids. While MUFA contents (of 14:1n- 5, 16:1n- 7, and 18:1n- 9) were lowered in the pioglitazone group, the PUFA content (of 22:6n- 3, 20:3n- 6, 20:4n- 6, and 22:4n- 6) was increased. This suggests that the redistribution of lipids into adipose tissue, a well-understood mechanism of PPARγ agonists in humans and rodent models,[63] is associated with the depletion of non-esterified PUFAs from the liver and the serum in rats.

A similar, overall reduction of NEFAs in plasma following pioglitazone treatment has been observed in mice.[23] It is also noteworthy that in the same study, a combined administration of pioglitazone and PUFAs augmented the positive effects of the drug alone on plasma lipid profile and glucose homeostasis,[23] suggesting that a re-supplementation of PUFAs following their depletion induced by disease and the TZD’s is beneficial.[64]

Surprisingly, the decrease in NEFAs was not accompanied with a concomitant decrease in glycerol levels, which remained constant in the liver and the intestine, and were even elevated in serum. This indicates that the removal of glycerol by lipogenesis is superseeded by the resupply of glycerol by de novo biosynthesis. It illustrates a limitation of the present study, that does not captured quantitative rates of metabolite anabolism and catabolism with its single timepoint snapshot measurement. For this purpose, a targeted fluxome analysis using stable isotope-labeled precursors appears appropriate in future studies.[65]

The levels of 3-hydroxybutyrate in the serum of pioglitazone-treated animals were found to be decreased to 63% of the level found in untreated animals. This probably reflects the resensitization to insulin, as with the improved glucose disposal, the pressure to catabolize fatty acids via β-oxidation is decreased. Although the activation of PPARs is known to trigger β-oxidation,[66,67] transcriptional profiles in the adipose tissue of db/db mice suggest that this process is less pronounced with pioglitazone, which rather activates lipid deposition by increasing lipid synthesis and transport.[68]

### Amino acid profiles

The obese diabetic rat experienced decreased levels of amino acids in particular in the liver (see above and Tables 13 and 14). The daily pioglitazone treatment over 13 weeks significantly increased the abundance of both glucogenic and ketogenic amino acids to levels of the non-diabetic group in the liver. We interpret this elevation in the level of glucogenic amino acids following pioglitazone treatment as a signature of inhibition of hepatic gluconeogenesis [53,69–71]. The changes observed in the serum of drug-treated animals were more differentiated: While the levels of many amino acid like Phe, Tyr, Lys, Cys, Met, Gly or Ser rose, the levels of BCAA’s isoleucine, leucine and valine decreased further. Likewise, the levels of glutamate, a byproduct of BCAA catabolism, and of the catabolic intermediates 3-methyl-2-oxobutyrate, 3-methyl-2-oxovalerate, or 2-hydroxyisobutyrate dropped.

**Table 13:** Concentration ratios of amino acids in the serum (the color code is defined in the materials and methods section).

Serum	Obese (NC)/ Lean (NC)	Pio (HF)/ Obese (HF)	Obese (NC) / Lean (NC)		Pio (HF) / Obese (HF)	
Biochemical			p-value	q-value	p-value	q-value
glycine	1,2	**2,11**	0,2532	0,1373	0,0005	0,002
serine	0,96	**1,56**	0,7608	0,3299	0,0157	0,0221
threonine	**0,67**	0,96	0,0399	0,0323	0,5639	0,2835
aspartate	0,82	0,72	0,3715	0,1883	0,1547	0,1135
asparagine	**1,31**	1,16	0,0035	0,0049	0,2403	0,1591
alanine	**0,75**	1,05	0,0907	0,0615	0,9218	0,3811
glutamate	0,96	**0,55**	0,7895	0,3396	0,0211	0,026
glutamine	0,86	**1,23**	0,3088	0,162	0,0987	0,0812
histidine	**0,86**	**1,41**	0,0455	0,0359	0,0556	0,053
lysine	**0,44**	**1,58**	0,0042	0,0056	0,0203	0,0256
phenylalanine	**0,86**	**1,34**	0,0106	0,0113	0,014	0,021
tyrosine	1,05	**1,66**	0,92	0,3771	0,0097	0,0156
tryptophan	**0,81**	**0,79**	0,0262	0,0227	0,0832	0,0728
isoleucine	**1,44**	**0,51**	0,0006	0,0013	1,90E-05	0,0002
leucine	**0,83**	**0,42**	0,0575	0,0421	3,32E-06	6,75E-05
valine	**1,12**	**0,66**	0,0934	0,0626	7,45E-05	0,0005
cysteine	**0,72**	**2,18**	0,0172	0,0161	4,26E-05	0,0004
methionine	**0,64**	**1,2**	8,70E-05	0,0003	0,0148	0,0211
arginine	**0,74**	**1,41**	0,0967	0,064	0,0842	0,0733
proline	**0,72**	**0,8**	0,0008	0,0016	0,0545	0,0528

**Table 14:** Concentration ratios of amino acids in the liver (the color code is defined in the materials and methods section).

Liver	Obese (NC)/ Lean (NC)	Pio (HF)/ Obese (HF)	Obese (NC) / Lean (NC)		Pio (HF) / Obese (HF)	
Biochemical			p-value	q-value	p-value	q-value
glycine	0,9	**1,63**	0,1975	0,1326	0,0018	0,0098
serine	**0,64**	**2,56**	0,0012	0,0027	3,12E-05	0,0016
threonine	**0,5**	**1,66**	0,0023	0,0043	0,0084	0,0232
aspartate	**0,68**	**1,59**	0,0397	0,0406	0,0371	0,0645
asparagine	**0,58**	**2,19**	0,0334	0,0353	0,0002	0,0038
alanine	0,9	1,1	0,1875	0,1264	0,5553	0,3393
glutamate	1,08	**1,52**	0,4994	0,265	0,0033	0,014
glutamine	**0,64**	**1,51**	0,0003	0,0011	0,0026	0,012
histidine	**0,79**	**1,28**	0,002	0,004	0,014	0,0335
lysine	**0,82**	1,12	0,0003	0,0011	0,1686	0,1714
phenylalanine	**0,59**	**1,46**	2,81E-05	0,0002	0,0201	0,0424
tyrosine	**0,67**	**1,49**	0,0006	0,0016	0,0076	0,0219
tryptophan	**0,59**	**1,38**	1,09E-05	0,0001	0,0097	0,0252
isoleucine	**0,63**	**1,24**	0,0003	0,001	0,0921	0,1145
leucine	**0,61**	**1,26**	1,05E-05	0,0001	0,0142	0,0336
valine	**0,69**	**1,3**	4,26E-05	0,0003	0,0316	0,0583
cysteine	0,85	**1,44**	0,1304	0,0985	0,0443	0,0722
methionine	**0,68**	**1,41**	1,99E-05	0,0002	0,0071	0,0215
arginine	0,65	**3,73**	0,1309	0,0985	0,0404	0,0686
proline	**0,71**	**1,22**	1,04E-05	0,0001	0,09	0,1132

These observations are in line with the finding that PPARγ agonists strongly upregulated catabolic BCAA enzymes in adipose tissue [72,73]. The reduction of BCAA’s and of glutamate has also been reported in human intervention studies with TZD’s[74,75], and a similar, but non-significant trend was captured in a study of pioglitazone effects in C57BL/6N mice under high fat diet[23]. Also the increase of Ser and Gly was noticed in a human study with adults with impaired fasting glucose or untreated diabetes who were treated with metformin and pioglitazone, but in contrast to our findings, the aromatic acids Phe, Tyr, Lys, or Arg decreased in concentration.[28]

In the intestine, BCAA levels were unaltered; however, short chain acyl-carnitines including isobutyrylcarnitine, 2-methylbutyroylcarnitine and isovalerylcarnitine that are derived from catabolic intermediates were strongly decreased (S1 File, Table F).

Thus, the study confirms the reported association of insulin (re-)sensitization with reduced BCAA levels in the serum[42], but also highlights that the drug-induced metabolite changes are strongly tissue-dependent.

### Oxidative stress–Antioxidant response system

Enhanced oxidative stress in the diabetic state was indicated by elevated levels of oxidized glutathione (GSSG), cysteine-glutathione disulfide, and ophthalmate, as outlined above. In contrast to metabolite classes discussed in the previous sections, the levels of oxidative stress markers in serum were not re-adjusted to normal levels observed for nondiabetic rats, but further enhanced 2.3-, 1.9-, and 2.8-fold upon pioglitazone treatment, respectively (S1 File, Table B). This finding is surprising in view of several studies that reported an enhanced activity and/or expression of oxidative stress response markers (e.g. superoxide dismutase SOD) upon PPARγ activation with TZD’s.[76–79] On the other hand, He *et al*. reported that mitochondrial oxidative stress in mouse hearts was induced by rosiglitazone by a PPAR-independent mechanism.[80] A PPAR-independent effect could also be demonstrated by the suppression of ferroptosis mediated by the oxidative stress enzyme GSH peroxidase 4 (GPX4).[81] Instead, the inhibition of acyl-CoA synthetase long-chain family member 4 (ACSL4) by TZD’s[82] was proven to be responsible for the anti-ferroptotic effect. However, a direct comparison with our findings is difficult, as different markers have been investigated in the studies cited above.

## Conclusions

In summary, an in-depth analysis of metabolic changes associated with genetic, nutritional and pharmacological perturbations in the female ZDF rat was conducted using mass spectrometry. All perturbations induced pronounced shifts in metabolism that were consistent with the phenotype observed by established clinical biomarkers and an in vivo imaging analysis. The diabetic state in obese rats was associated with hyperglycemia and decreased amino acids levels, reflecting stimulation of hepatic gluconeogenesis, dyslipidemia and elevated β-oxidation. As mass spectrometry achieves a resolution to single components rather than capturing whole classes, subtle differences in metabolite profiles, e.g. between short chain, long chain and polyunsaturated fatty acids, or between amino acid subclasses could be detected. Most of the biochemical signatures of diabetes were reversed by pioglitazone, underlining the broad impact of PPARγ activation that is overall beneficial for metabolic parameters. With 420 biochemicals in serum, 443 in liver and 603 in intestine, the study represents the most detailed metabolomic investigation of the ZDF rat model and of TZD effects reported so far. It deepens the understanding of the ZDF rat model and provides an extensive dataset that can serve to pinpoint similarities and differences on the metabolite level between the ZDF rat and humans with diabetes. Overall, the metabolite alterations in major biochemical pathways further validate the ZDF rat as a suitable preclinical model of diabetes in humans. For an enhanced understanding of the observed change patterns, a system-oriented view, built by linking metabolite levels with the transcriptional regulation of their biosynthesis and catabolism might be a fruitful extension for future studies.

## Supporting information

S1 File Supplementary data tables. The Tables include MS intensity data of all metabolites in individual animals from all matrices (tabs serum data/liver data/intenstine data). They also include a statistical analysis of the fold changes of metabolite abundances between groups, including statistical information. (tabs Serum Stats/Liver Stats/Intestine Stats). In the statistical analysis, the metabolites are grouped according to biochemical pathways.
(XLSX)
